# Extended haplodiploidy hypothesis

**DOI:** 10.1002/evl3.119

**Published:** 2019-04-29

**Authors:** Petri Rautiala, Heikki Helanterä, Mikael Puurtinen

**Affiliations:** ^1^ School of Biology University of St Andrews St Andrews KY16 9TH United Kingdom; ^2^ Organismal and Evolutionary Biology Research Programme University of Helsinki PO Box 65 FI‐00014 Finland; ^3^ Centre of Excellence in Biological Interactions; ^4^ Ecology and Genetics Research Unit University of Oulu POB 3000 FI‐90014 Finland; ^5^ Department of Biological and Environmental Science University of Jyvaskyla PO Box 35 FI‐40014 Finland

**Keywords:** Haplodiploidy hypothesis, inclusive fitness, kin selection, reproductive altruism

## Abstract

Evolution of altruistic behavior was a hurdle for the logic of Darwinian evolution. Soon after Hamilton formalized the concept of inclusive fitness, which explains how altruism can evolve, he suggested that the high sororal relatedness brought by haplodiploidy could be why Hymenopterans have a high prevalence in eusocial species, and why helpers in Hymenoptera are always female. Later it was noted that in order to capitalize on the high sororal relatedness, helpers would need to direct help toward sisters, and this would bias the population sex ratio. Under a 1:3 males:females sex ratio, the inclusive fitness valuation a female places on her sister, brother, and an own offspring are equal—apparently removing the benefit of helping over independent reproduction. Based on this argumentation, haplodiploidy hypothesis has been considered a red herring. However, here we show that when population sex ratio, cost of altruism, and population growth rate are considered together, haplodiploidy does promote female helping even with female‐biased sex ratio, due the lowered cost of altruism in such populations. Our analysis highlights the need to re‐evaluate the role of haplodiploidy in the evolution of helping, and the importance of fully exploring the model assumptions when comparing interactions of population sex ratios and social behaviors.

**Impact summary**

Kin selection theory explains how traits that are harmful for the actor's own fitness can be selected for when copies of the underlying genes are passed to future generations by relatives benefiting from the actor's altruistic behavior. Soon after William Hamilton formalized the concept of inclusive fitness, which accounts the actor's effects on the fitness of its relatives, he suggested that altruistic behavior would evolve more easily in Hymenoptera (ants, bees, and wasps) due to their haplodiploid sex determination system—a system not shared by, for example, mammals. In haplodiploid taxa, females are born from fertilized and males from unfertilized eggs—females have therefore two sets of genes, whereas males have only one. Since males always pass on the same genes to their daughters, full sisters are more related to each other than a female is to her own offspring. In hymenopteran colonies helpers are always female, an expected aspect of this “haplodiploidy hypothesis.” Having been presented in the same papers with inclusive fitness theory, haplodiploidy hypothesis became a showpiece for inclusive fitness thinking. When the haplodiploidy hypothesis was later suggested to be theoretically flawed, some even thought this meant that inclusive fitness thinking had no merit. Because of this historical connection, and its value as an introduction to inclusive fitness thinking, the logic and failure of haplodiploidy hypothesis are presented in modern textbooks explaining inclusive fitness. However, here we show that the dismissal of haplodiploidy hypothesis has been premature. By detailing the relationship between cost of altruism, population sex ratio, and population growth rate—something the previous models missed—we show that haplodiploidy indeed promotes the evolution of female helper castes.

Sterile worker castes in taxa such as ants and social bees and wasps seem problematic for the logic of Darwinian evolution. The problem was solved formally by Hamilton (1964) as a part of his theory of inclusive fitness. A gene responsible for reproductive altruism can be selected for if an actor can transmit more of its copies to future generations by helping relatives than by pursuing own reproduction. This condition is often expressed as

(1) $$br_{b}>cr_{c}$$

where *b* denotes the number of relatives reared by helping, *r*_b_ the inclusive fitness valuation of relatives to the actor, *c* the expected number of own offspring sacrificed by not reproducing, and *r*_c_ the inclusive fitness valuation of own offspring to the actor (Hamilton, 1964, 1972; West‐Eberhard 1975). Rearranging, the required number of relatives raised for helping to be selected for (*b*_tr_) can be expressed as

(2) b tr :=rcrbc<b.

Hamilton famously suggested that the high prevalence of eusociality with female helpers in Hymenoptera could be explained by their peculiar haplodiploid sex‐determination mechanism where haploid males are born from unfertilized eggs and diploid females from fertilized eggs (Hamilton, 1964, 1972). Under monogamous haplodiploidy, a female is more related to her full sisters than to her own offspring. Hamilton suggested that this would predispose haplodiploid females to altruistic helping, explaining why reproductive altruism was so common in haplodiploids and why helpers in social Hymenoptera are always female. This suggestion became to be known as the “haplodiploidy hypothesis” (Hamilton, 1964, 1972; West‐Eberhard 1975).

However, the haplodiploidy hypothesis was soon found to have several weaknesses. Although a female is highly related to her sisters, she is less related to her brothers. Trivers and Hare (1976) noted that in a population with an even sex ratio, an average sibling is no more valuable to a female than an own offspring, and haplodiploidy thus does not promote indiscriminate helping. They concluded that in order to capitalize on the high sister‐sister relatedness, a female would need to direct her helping toward sisters. Help directed to sisters would however result in the population sex ratio becoming female‐biased, until it reaches 1:3 ratio of males to females (Trivers and Hare 1976). At this population sex ratio, the inclusive fitness valuations a haplodiploid female places on her brother, sister, and average offspring are equal, and haplodiploidy again does not seem to promote helping (Trivers and Hare 1976; Charnov 1978; Craig 1979; Grafen 1986). This refutation of the haplodiploidy hypothesis is widely used as an example of inclusive fitness thinking (Bulmer 1994; Bourke and Franks 1995; Smith and Szathmáry 1997; Davies et al. 2012; Marshall 2015; Dawkins 2016).

In this paper, we show that the dismissal of the theoretical basis of haplodiploidy hypothesis has been premature. Previous studies (e.g., Trivers and Hare 1976; Grafen 1986) restricted their analysis to inclusive fitness valuations (terms *r*_c_ and *r*_b_ in Hamilton's rule), without considering the effects of helper‐manipulated sex ratio on population growth rate and the cost of altruism (term *c* in Hamilton's rule). We incorporate population growth rate to inclusive fitness calculations, and find that when comparing populations with equal growth rate—stable sized populations being the most biologically relevant special case—haplodiploidy promotes the evolution of female helpers also in female‐biased populations. Our analysis suggests that the role of haplodiploidy in the evolution of helping should be reconsidered.

## Methods and Results

### MODEL DESCRIPTION

We obtain the required number of relatives a helper needs to rear for helping to be more beneficial than direct reproduction in both haplodiploid and diplodiploid populations. This is done for both males and females who either rear siblings according to the population sex ratio, or focus their helping efforts on brothers or sisters only. We assume weak selection, so that the future expectations for each same sex juvenile pursuing own reproduction are equal. Both sexes cost the same to produce, making sex allocation and sex ratio synonymous. Inclusive fitness valuations are assumed to be equal for all relatives belonging to the same class (brothers, sisters, sons, daughters). All mentions of sex ratios (*z*, proportion of males; see Table 1 for all variables and their definitions) exclude helpers, and each nest produces offspring with the population sex ratio. Changes in the sex ratio during the lifespan of a single individual is assumed to be negligible, even if changing significantly over evolutionary time. The analysis holds for monogamous and panmictic populations with either non‐overlapping generations, or with overlapping generations with discrete cohorts. The population can start out as the former and evolve into the latter as helping evolves. Cohort size refers to the total number of non‐helper juveniles produced to each discrete cohort. A helper can rear siblings to any cohort(s) where its mother is reproductively active.

**Table 1:** Definitions of variables

Symbol	Definition
*b*	total number of relatives reared by a helper
$b_{k}$	number of relatives reared to cohort *k* when the actor belongs to cohort 0, $\sum_{k=0}b_{k}=b$
*c*	expected number of offspring lost by becoming a helper
$c_{k}$	expected number of juvenile offspring lost from cohort *k* when the actor belongs to cohort 0, $\sum_{k=1}c_{k}=c$
*r*_b_	inclusive fitness valuation of an average reared relative
*r*_c_	inclusive fitness valuation of an average offspring lost
*b*_tr_	minimum number of relatives reared for helping to be beneficial
*z*	proportion of males among non‐helper juveniles
*m*	number of non‐helper juveniles belonging to the actor's cohort
λ	the cohort size is multiplied by this scalar each successive cohort, λ=1 for a stable sized population
*p*_relative_	consanguinity between the actor and its respective relative
*v*_f_ and *v*_m_	class reproductive values of juvenile females and males respectively, $v_{f}=2v_{m}$ for haplodiploids and $v_{f}=v_{m}$ for diplodiploids
*z*_o_	expected offspring production sex ratio, $z_{o}=0$ for haplodiploid males and $z_{o}=z$ for other actors
*z*_h_	how helping is administered: $z_{h}=z$ when helping is directed to brothers and sisters according to the population sex ratio;$\;z_{h}=0$ when directed at sisters; and $z_{h}=1$ when directed at brothers

**Figure 1:**
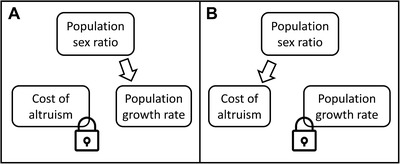
The population sex ratio, population growth rate, and the cost of altruism (*c*‐term in the Hamilton's rule, i.e., the number of offspring a juvenile expects to produce) are all interconnected, so that changing one and keeping the other two constant is impossible in a homogenous population (see eqs. (3), (4), (5)). (A) In previous analyses of the haplodiploidy hypothesis, the cost of altruism was assumed to remain constant when population sex ratio changes from 1:1 to 1:3 males:females. However, our analysis shows that a shift in population sex ratio induces a shift in population growth rate, which is not sustainable on evolutionary time scales. (B) A biologically more plausible assumption is an approximately constant population growth rate, and especially an approximately constant population size. In this case, a shift in population sex ratio induces a shift in the cost of altruism (as has been pointed out for example by Gardner and Ross 2013 and Davies et al. 2016). This shift in the cost of altruism brings about the lowered benefit threshold of helping for haplodiploid females also in female‐biased population sex ratios.

To incorporate population growth to the analysis, we assume that the cohort size is multiplied by λ each successive cohort during a single individual's lifespan (λ=1 for a stable sized population). This scalar can however change over evolutionary time. When we compare conditions for helping to evolve, we assume that λ is equal in the compared scenarios (e.g., when comparing haplodiploid and diplodiploid females). Previous analyses have not considered how population growth affects cost of altruism and inclusive fitness valuations (Fig. 1).

### THE COST OF ALTRUISM

The cost of altruism *c* is the number of offspring a non‐helper juvenile expects to gain in its lifetime. When the population is not stable but grows or shrinks, the inclusive fitness valuation placed on a single relative depends on the cohort it belongs to. Therefore, for our purposes the cost of altruism *c* needs to be partitioned into cohort specific quantities with appropriate weights to account for the cohort‐specific value of relatives. A juvenile individual expects to produce *c*_1_ offspring to the following cohort, *c*_2_ offspring to the cohort after that and so forth. The cost of altruism is, by definition, the sum of these quantities, $c=\sum_{k=1}c_{k}$ (in a univoltine and semelparous population only *c*_1_ differs from zero). The exact numerical quantities can be derived only for a stable sized population (λ=1), but the general equalities we derive below (eqs. (3), (4), (5)) can be substituted later in the inclusive fitness inequality 7 to replace difficult terms.

First, we find the cost of altruism for haplodiploid and diplodiploid females. Consider a population with proportion of *z* males and cohort size *m*. That cohort is produced by $\left(1-z\right)\frac{m}{\lambda }$ females born in the previous cohort each expecting to produce *c*_1_ offspring to the focal cohort, along with $\left(1-z\right)\frac{m}{\lambda^{2}}$ females born two cohorts earlier, each expecting to produce *c*_2_ offspring to the focal cohort and so forth. The sum of these expectations, by definition, equals the size of the focal cohort: $\sum_{k=1}\left(1-z\right)\frac{m}{\lambda^{k}}c_{k}=m$. By reorganizing, we have that the partitioned cost of altruism for both haplodiploid and diplodiploid females satisfies

(3) $$\sum_{k=1}\frac{c_{k}}{\lambda^{k}}=\frac{1}{1-z}.$$

For diplodiploid males, the similarly derived link between offspring production and focal cohort size is $\sum_{k=1}z\frac{m}{\lambda^{k}}c_{k}=m$, from which we have that the partitioned cost of altruism for a diplodiploid male satisfies

(4) $$\sum_{k=1}\frac{c_{k}}{\lambda^{k}}=\frac{1}{z}.$$

Finally, because haplodiploid males from previous cohorts are only fathering the (1−z)m females of the focal cohort, the link between offspring production and focal cohort size is $\sum_{k=1}z\frac{m}{\lambda^{k}}c_{k}=\left(1-z\right)m$, from which we have that the partitioned cost of altruism for a haplodiploid male satisfies

(5) $$\sum_{k=1}\frac{c_{k}}{\lambda^{k}}=\frac{1-z}{z}.$$

These weighted sums for the partitioned cost of altruism for all possible actors depend only on the population sex ratio and population growth rate, not on the expected fecundity of successfully reproducing individuals or on cohort size. Therefore, under weak selection these equations hold for all stages of helper evolution, as do the subsequent inclusive fitness conditions. In stable sized populations (λ=1), the left sides of equations (3), (4), (5) equal the cost of altruism *c*, which highlights the effect population sex ratio has on the actual cost of sacrificing the pursuit of own reproduction.

### THE INCLUSIVE FITNESS VALUATIONS

While *b* and *c* capture the number of relatives gained and lost by becoming a helper, the inclusive fitness valuations *r*_b_ and *r*_c_ give the value for a single individual belonging to either category. The inclusive fitness valuation the actor places on its kin equals the individual reproductive value of the target, which represents how that individual contributes to the future genepool (Taylor 1996; Taylor and Frank 1996), multiplied by the consanguinity between the actor and the target (*p*_relative_; a measure of relatedness; Bulmer 1994). We consider an age‐structured population and only focus on one age group: the juveniles. The individual reproductive value of a single juvenile is arrived at by dividing the total reproductive value of the class it belongs to, that is, either juvenile females (*v*_f_) or juvenile males (*v*_m_), with the number of same sex juveniles belonging to its cohort.

It is also necessary to account for the effects of population growth on inclusive fitness losses and gains. We do this by partitioning the inclusive fitness expectations for pursuing own reproduction cohort by cohort, as well as the inclusive fitness gains when becoming a helper. As a helper, an individual would rear *b*_0_ siblings to its own cohort (relevant for univoltine and semelparous populations), *b*_1_ siblings to the next cohort and so forth. The general form of the Hamilton's rule for an individual belonging to a focal cohort of size *m* after this partitioning becomes

(6) ∑k=1ckzop son vmzλkm+1−zop dau vf1−zλkm<∑k=0bkzhp bro vmzλkm+1−zhp sis vf1−zλkm,

where *z*_o_ is the expected offspring production sex ratio ($z_{o}=0$ for haplodiploid males and $z_{o}=z$ for other actors due to weak selection) and *z*_h_ represents how helping is administered ($z_{h}=z$ when helping is directed to brothers and sisters according to the population sex ratio;$\;z_{h}=0$ when directed at sisters; and $z_{h}=1$ when directed at brothers). By first taking $\lambda^{k}$ terms as common factors within both sums, multiplying both sides by $z\left(1-z\right)m/v_{m}$, and finally reorganizing, we attain that for helping to be beneficial it must satisfy inequality

(7) p son zo1−z+p dau z1−zovfvmp bro zh1−z+p sis z1−zhvfvm∑k=1ckλk<∑k=0bkλk.

In haplodiploids, the class reproductive value of juvenile females is double that of juvenile males ($v_{f}/v_{m}=2;\;$Gardner 2014) whereas in diplodiploids, the class reproductive values of juvenile females and males are equal ($v_{f}/v_{m}=1$; Grafen 2014). These relative values hold when the class distributions remain stable, which is true for our model due to the stable fractional change in the successive cohort sizes. Under panmictic monogamous haplodiploidy, the consanguinities between sisters is 3/8; between a female and her brother 1/4; between brothers 1/2; between a mother and her daughter 1/4; between a mother and her son 1/2; and between a father and his daughter 1/2 (Bulmer 1994). Under diplodiploidy all these consanguinities, together with the one between a father and his son, equal 1/4 (Bulmer 1994).

### THE BENEFIT THRESHOLD *b*_tr_

For helping to be favored, the number of extra relatives accrued by helping must be larger than the benefit threshold (b tr :=rcrbc; eq. (2)). By comparing the benefit thresholds *b*_tr_ for different actors, targets, sex‐determination systems, and population sex ratios, it is possible to see under which circumstances helping evolves most easily (i.e., under which circumstances the benefit threshold is the lowest). However, as the partitioned Hamilton's rule shows (right side of inequality 7), the benefit terms are weighted by the proportion how cohort size changes between the respective sibling's cohort and that of the actor.

By substituting in the corresponding consanguinities, reproductive value relations, expected offspring sex ratios, and, most importantly, the respective cost equation (eqs. (3), (4), (5)) into inequality 7, we have the benefit threshold for a haplodiploid female as

(8) b tr :=43−zh4−1z<∑k=0bkλk;

for a diplodiploid female as

(9) b tr :=21−zh2−1z<∑k=0bkλk;

and for both haplodiploid and diplodiploid male as

(10) b tr :=21−1−zh2−11−z<∑k=0bkλk.

where *z*_h_ represents the mode of helping and is equal to 0 when directed at sisters, equal to 1 when directed at brothers, and equal to the population sex ratio *z* when helping is administered according to population sex ratio.

The form of expressions in inequalities 8–10 make two things evident. First, when population sex ratio is 1/4 for haplodiploid females or 1/2 for other actors, brothers and sisters are equally valuable to the helper (the terms involving *z*_h_ cancel out). This result was derived by Trivers and Hare (1976). Second, the population sex ratio has no effect on the benefit threshold when rearing same sex siblings (the terms involving *z* cancel out). This novel result highlights the importance of considering the effects of sex ratio on both inclusive fitness valuations and costs when analyzing the evolutionary origin of altruism.

**Figure 2:**
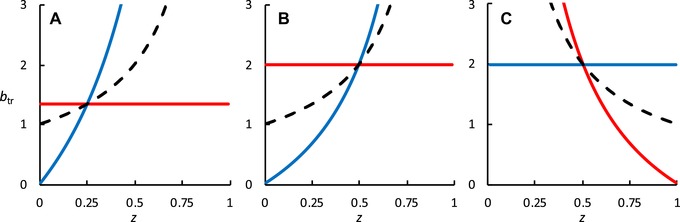
The benefit thresholds of helping for (A) haplodiploid female, inequality 8; (B) diplodiploid female, inequality 9; (C) male under either haplodiploidy diplodiploidy, inequality 10, as a function of population sex ratio (*z*). In each panel, red line presents helping sisters, blue line presents helping brothers, and dashed line presents helping administered according to the population sex ratio ($z_{h}=0$,$\;z_{h}=1$, and $z_{h}=z$, respectively). Helpers have a selection pressure to bias their helping toward the sex with the lower benefit threshold until the population sex ratio shifts so that the benefit thresholds are equal. Haplodiploid female helpers will thus, if able, shift the population sex ratio to z=0.25. At this equilibrium point, the benefit threshold for any type of helping is 4/3. Diplodiploid female helpers and male helpers would be selected to keep the population sex ratio at z=0.5, and the benefit threshold for helping is two.

The benefit thresholds (inequalities 8–10) are presented for each mode of helping and possible actor in Figure 2. In a stable sized population (λ=1), the thresholds give a simple numerical answer to the question “How many siblings a helper needs to rear for helping to be beneficial?” The right side of inequalities 8–10 lose the weights from the sum terms and become purely the number of relevant siblings reared $\left(\sum_{k=0}b_{k}=b\right)$. The values in Figure 2 can thus be interpreted directly as the number of siblings a helper must produce in order for helping to be favored in a stable sized population. The same loss of weights happens for the cost equations (3), (4), (5), and these equations then represent the expected absolute number of offspring lost by not pursuing independent reproduction.

As can be seen from Figure 2, haplodiploid females need to produce 4/3 sisters in a stable sized population for helping to be beneficial, regardless of the population sex ratio or general fecundity of the taxa. Furthermore, under 1:3 males:females sex ratio, the required number is the same (4/3), regardless of the sex of the produced siblings. This contrasts with the required number for diploid males and females and for haplodiploid males, who would need to produce more than two siblings at population sex ratio 1:1. Thus, the efficiency requirement for the evolution of helping is considerably lower for haplodiploid females than for other types of helpers at all relevant population sex ratios. To verify these analytical results, we have built population genetic simulations to study invasion success of helping alleles at different population sex ratios and at different helping efficiencies (see Supporting Information; Tables S1 and S2). The outcomes of the simulations align with the analytical results presented in Figure 2.

Also in the case of a univoltine semelparous population, where nests are founded by new queens each year, the analysis yields simple numerical results. In this case, the right hand side of inequalities 8–10 becomes the number of siblings reared to the actor's cohort (*b*_0_), without cohort‐specific weights. Thus, the values in Figure 2 can also be interpreted as the absolute number of siblings a helper must produce in order for helping to be favored in a univoltine semelparous population, regardless of the growth trajectory of the population.

## Discussion

We have shown that the efficiency requirement for evolution of reproductive altruism is lower for haplodiploid females than for other types of actors, irrespective of population sex ratio. This contrasts with previous analyses, which have concluded that haplodiploidy does not promote altruism in highly female biased populations outside specific scenarios based on split sex ratios (e.g., Trivers and Hare 1976; Craig 1980; Grafen 1986; Bulmer 1994; Bourke and Franks 1995; Smith and Szathmary 1997; Davies et al. 2012; Gardner et al. 2012; Marshal 2015; Dawkins 2016; Quiñones and Pen 2017). Due to the relatedness asymmetries arising from haplodiploidy, a female is more related to her full sisters than to her own offspring (Hamilton, 1964, 1972; Trivers and Hare 1976). We have shown that helping is selected for if a female helper can raise just over 4/3 full sisters at 1:1 population sex ratio in a stable sized population (inequality 8), whereas all other types of actors would need to raise more than two siblings. Furthermore, when the helpers shift the population sex ratio toward females, the need for helpers to discriminate between brothers and sisters disappears. Under 1:3 males:females population sex ratio, a female helper still needs to produce only just over 4/3 full siblings (of either sex) for helping to be selected for. Thus, haplodiploidy provides consistent selective advantage for evolution of female helper castes that is not present for any other type of actor (diplodiploid males or females, or haplodiploid males).

Two crucial assumptions we make are monandrous mating, which guarantees a high sororal relatedness, and the ability for helpers to treat sisters and brothers differently. Monandry was the ancestral state of the lineages that evolved eusociality (Hughes et al. 2008), and is thus a reasonable model assumption. Worker‐controlled sex ratios seem common in social Hymenoptera (Meunier et al. 2008), including primitively social species (Boomsma and Eickwort 1993; Packer and Owen 1994), and adaptive sex ratio manipulation by helpers in primitively social bees (Mueller 1991) as well as ants (Sundström 1994) has been demonstrated. Thus, ancestral presence of sex biased behaviors by helpers is a plausible scenario.

The key difference between previous models and the current analysis is in how population growth rate is taken into consideration in the model, if at all. The current analysis reveals that population sex ratio always has an effect on either the cost of altruism or on population growth rate (Fig. 1). When inclusive fitness valuations of siblings and own offspring have been compared in previous analyses at different sex ratios (e.g., Trivers and Hare 1976; Grafen 1986), the implicit assumption has been that the sex ratio does not influence the cost of altruism (i.e., the number of offspring lost when becoming a helper). However, this assumption means that in a population with a female‐biased sex ratio each female expects to produce more daughters than in a population with an even sex ratio. Those daughters in turn would expect to produce more daughters of their own, resulting in an exponentially growing population (assuming that the population size is stable at even population sex ratio). Of course, exponentially growing populations are not sustainable over evolutionary timescales, and it is more relevant to compare inclusive fitness valuations in populations that are approximately stable in size.

The effect of population sex ratio on the cost of altruism has been identified in previous studies. Gardner and Ross (2013) and Davies et al. (2016) showed that if there is an initial bias between males and females in their ability to provide help, the mother should bias her primary sex ratio toward the more helpful sex. The biased sex ratio lowers the expected reproductive success of the more common sex (i.e., lowers the cost of altruism), creating a positive feedback between maternal sex ratio manipulation and altruistic helping. Our analysis differs from these models by showing that haplodiploidy promotes the evolution of female helpers also without initial bias in helping efficiency and without maternal sex‐ratio manipulation. We wish to stress that the models are not mutually exclusive, but offer different yet complementary viewpoints to understanding the evolution of altruism.

Female helpers in haplodiploid species are under selective pressure to bias helping toward sisters until the population sex ratio reaches 1:3 (Trivers and Hare 1976). Our analytical results (Figure 2) and population genetic simulations in the Supporting Information (Tables S1 and S2) show that helping evolves more easily in haplodiploid females than in other types of actor also under a female‐biased population sex ratio. Crucially, under a 1:3 sex ratio, the low benefit threshold applies to both brothers and sisters of haplodiploid females. This sets the stage also for relatively easy evolution of helping phenotypes that do not influence, or depend on, the sex of produced relatives. Even if the original helper phenotype would have been a brood carer and thus in a position to manipulate brood sex ratio, also traits not directly related to brood care, such as foraging and colony defense, could easily evolve under biased sex ratios. This may partly explain the large diversity of tasks carried out by social Hymenoptera workers, and even the evolution of specialized defensive casts in ants and stingless bees (Hölldobler and Wilson 2009; Grüter et al. 2017).

Other ways how haplodiploidy may promote helping could potentially work in concert with ours. With male‐biased dispersal, haplodiploidy promotes the evolution of helping among same‐generation females (Johnstone et al. 2011). When combined with monogamy, haplodiploidy promotes altruistic behavior via synergistic benefits (Fromhage and Kokko 2011). When potential female helpers are in nests that have a more female biased sex ratio than the population sex ratio, they experience an elevated in‐nest relatedness without the need to direct their helping efforts on their sisters. The importance of this “split sex ratios effect” (Grafen 1986) was questioned by Gardner et al. (2012) and Rautiala et al. (2014), but may nevertheless be play a role when combined with a bivoltine life cycle (Quiñones and Pen 2017) or adaptive virginity (Rautiala et al. 2018; this study also used the benefit threshold approach and a stable population size). Whether the insights of the current analysis would impact these other mechanisms is yet an open question. At least the conclusions of Rautiala et al. (2014) do not hold when population size is assumed to be stable and the effects of sex ratio on the cost of altruism are taken into account.

In summary, we have shown that haplodiploid female helpers have a lowered benefit threshold for helping regardless of the population sex ratio. We do not claim that this is the sole reason for the high prevalence of female helpers in haplodiploid organisms. However, when combined with favorable preadaptations such as maternal care (Queller, 1989, 1994; Gadagkar 1990; Queller and Strassmann 1998; Hunt 1999), the extended haplodiploidy hypothesis presented here suggests that haplodiploidy may indeed have contributed to the prevalence of reproductive altruism and to the rise of complex societies with multiple specialized female helper castes.

Associate Editor: K. Lythgoe

## Supporting information

Supplementary material for “Extended haplodiploidy hypothesis” by Petri Rautiala, Heikki Helanterä and Mikael Puurtinen.

**Table S1**. Dynamics of the allele coding for helping behaviour (allele value 1) in **haplodiploid** populations.
**Table S2**. Dynamics of the allele coding for helping behaviour (allele value 1) in **diplodiploid** populations.
