# Supplementary material for: Extended haplodiploidy hypothesis
Source: Evol Lett. 2019 Apr 29;3(3):263–70. doi: 10.1002/evl3.119 (PMC6546379; doi:10.1002/evl3.119)

**Supplementary material for “Extended haplodiploidy hypothesis” by Petri Rautiala, Heikki Helanterä and Mikael Puurtinen**

In addition to the analytical model presented in the main text, we built population genetic simulations to verify the main result that haplodiploidy promotes evolution of female sister helping also in female biased population sex ratios. We test the predictions for relevant combinations of ploidy level, sex of the helpers, primary sex ratios, and targets of helping efforts, as presented in Tables S1 and S2. For both haplodiploid and diplodiploid systems, we consider females rearing sisters, or both sisters and brothers, at an even sex ratio (1:1 M:F) and at a female biased sex ratio (1:3 M:F). In addition, we consider males rearing sisters and brothers at an even population sex ratio. For each considered case, we run simulations with the realized benefit of helping ($b$) being 80% and 120% of the predicted benefit thresholds ($b_{\mathrm{tr}}$). We find that the simulations fully support the predictions of the analytical model.

Simulation structure

In each simulation there are 600 nests producing offspring to two distinct cohorts. The number of cohorts is limited to two for simplicity. The population is divided to two asynchronous groups, group A and group B. When group A nests produce their second offspring cohort, Group B nests produce their first cohort, both of which are added into the mating pool (apart from the possible helpers in group B nests as explained below). Group A nests then die and are emptied, and are populated by a female and male in the mating pool to become the new parents of those nests. The individuals in the mating pool who didn't win a breeding position then die. In the next time step, the mating pool will consist of group B's second cohort and the newly formed group A's first cohort (again void of helpers as explained below), who will then compete for the group B's nest sites which are being emptied as group B nests die. This cycle continues throughout the simulation.

As a base reproduction, each nest produces 8 daughters and 8 sons to the first cohort and 20 daughters and 20 sons to the second cohort for even population sex ratio cases; and 12 daughters and 4 sons to the first cohort, and 30 daughters and 10 sons to the second cohort for 1:3 males:females population sex ratio cases. The simulation does not allow the primary sex ratio to evolve to avoid selection on the mother to invest more on the more helpful sex (Gardner & Ross 2013; Davies et al 2016), or for the mother to be able to return the primary sex ratio to even in the biased sex ratio cases.

Only the first cohort individuals can become helpers. A helper will never go into the mating pool, but instead rears $b$ (the realized benefit, explained below) new siblings to the second cohort. The efforts of the helpers in a single nest are pooled, and then rounded to a full number with appropriate probability. For example, if 3 helpers each rear 1.6 new sisters to the second cohort (totalling 4.8), the number of new sisters added to the mating pool is 5 with probability of 0.8 and 4 with probability 0.2.

Only first cohort individuals can become helpers. Whether an individual becomes a helper depends on its genotype in the helper-determining locus, with has alleles with values 0 or 1. For diploid individuals, the probability of becoming a helper is given by the average of the value of the two alleles (0, 0.5, or 1). Starting allele frequencies are set so that each simulation starts with 25% frequency of the allele that is predicted to be favoured by selection by the analytical model. Simulations are run until the locus is fixated for either allele. If there was no selection acting on the alleles, the *a priori* favoured allele would fix on 25% of runs by genetic drift. The carriers of these alleles are randomized for the first round. There are no mutations.

The simulations were run on Mathematica 11.3.0.0 and the notated files are attached. Fixed random seeds were used so the simulations can be replicated.

Results

Each case is was run 10 times. The null hypothesis is that without selection we would expect only 25% of the runs to end in the predicted allele to fixate. Each case was run for two different realized benefits $b$, 20% under and above the analytically derived benefit threshold (1.07 and 1.6 for a benefit threshold of 1.33; 1.6 and 2.4 for a benefit threshold of 2). As predicted, the results show that helping does not evolve under the analytical benefit threshold, and evolves above it (Tables S1 and S2).

Table S1. Dynamics of the allele coding for helping behaviour (allele value 1) in **haplodiploid** populations. The ‘Helper sex’ denotes the sex in which the helping allele is expressed; ‘Sex ratio’ denotes the primary sex ratio; ‘Reared siblings’ denotes the sex of reared siblings (‘Sisters and brothers’ are reared according to the primary sex ratio); ‘Predicted benefit threshold’ is the prediction from the analytical model (see main text); ‘Realized benefit of helping’ is the expected number of reared siblings per helper.


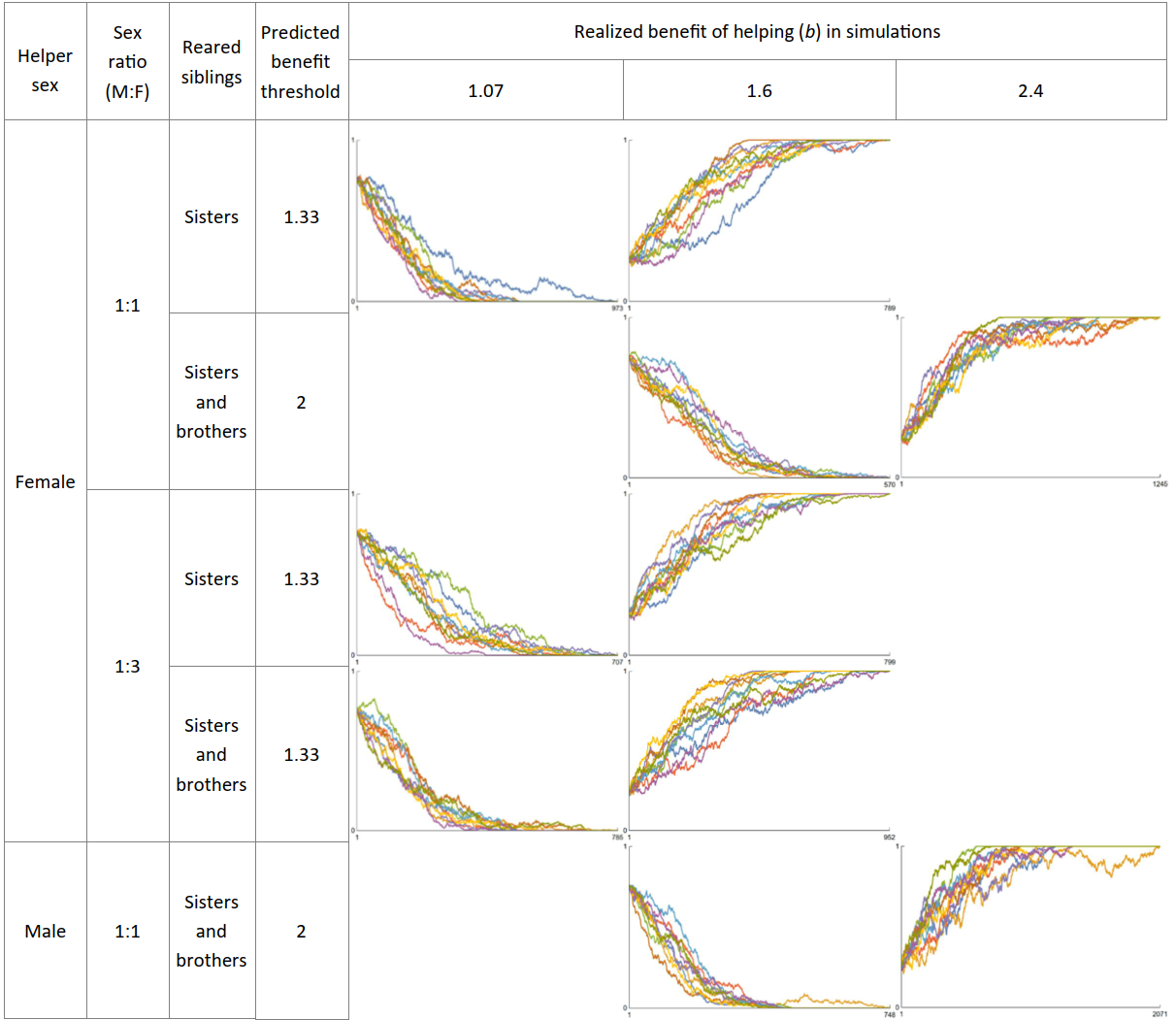


Table S2. Dynamics of the allele coding for helping behaviour (allele value 1) in **diplodiploid** populations. The ‘Helper sex’ denotes the sex in which the helping allele is expressed; ‘Sex ratio’ denotes the primary sex ratio; ‘Reared siblings’ denotes the sex of reared siblings (‘Sisters and brothers’ are reared according to the primary sex ratio); ‘Predicted benefit threshold’ is the prediction from the analytical model (see main text); ‘Realized benefit of helping’ is the expected number of reared siblings per helper.


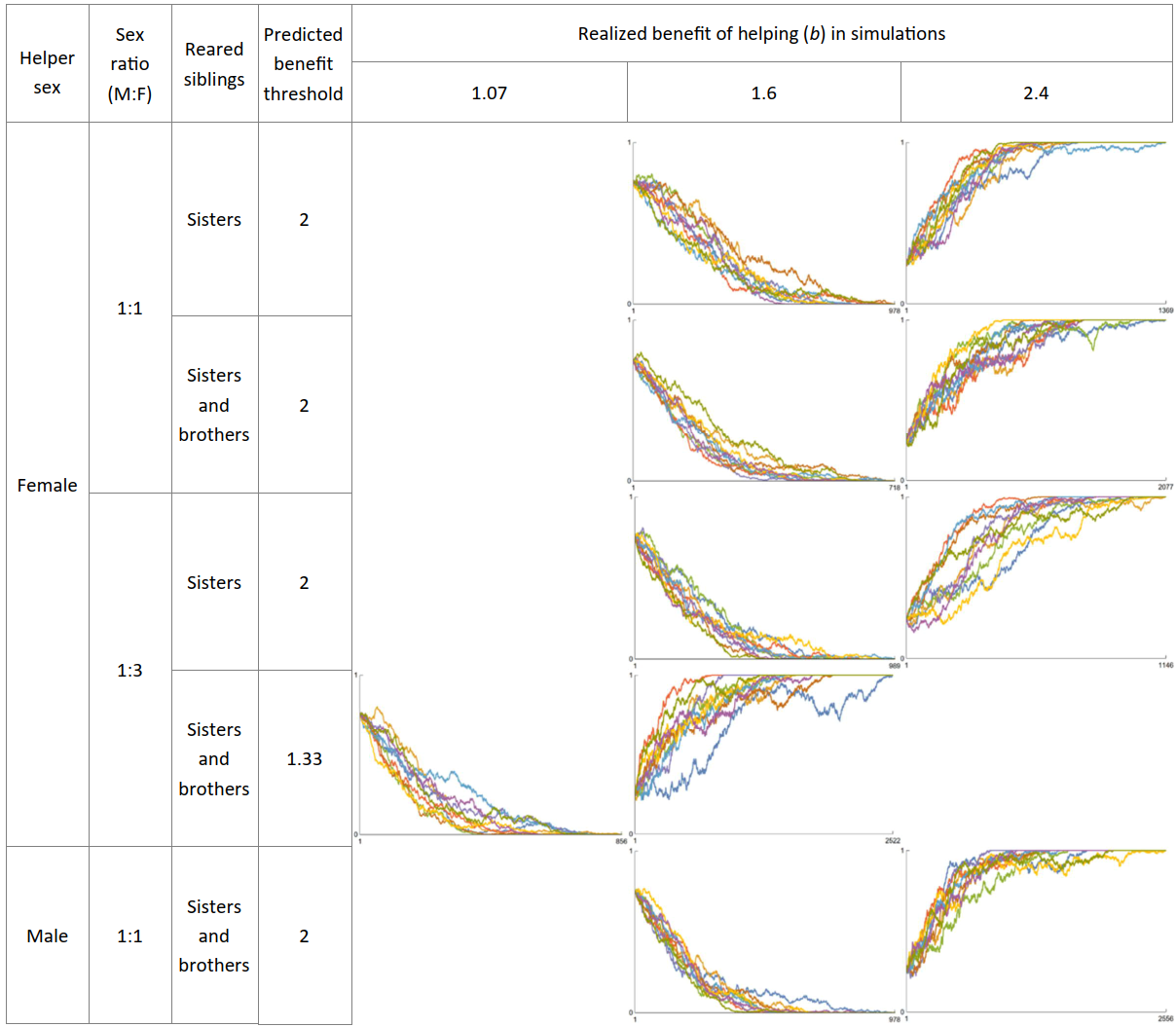

Supplement: Supplementary file 2 — Table S1. Dynamics of the allele coding for helping behaviour (allele value 1) in haplodiploid populations. Table S2. Dynamics of the allele coding for helping behaviour (allele value 1) in diplodiploid populations. [file EVL3-3-263-s002.docx]
